# Textures vs Non-Textures: A Simple Computational Method for Classifying Perceived ‘Texturality’ in Natural Images

**DOI:** 10.1177/20416695211054540

**Published:** 2021-10-28

**Authors:** Fumiya Kurosawa, Taiki Orima, Kosuke Okada, Isamu Motoyoshi

**Affiliations:** Department of Integrated Sciences, 13143 The University of Tokyo, Meguro-ku, Japan; Department of Life Sciences, 68394 The University of Tokyo Graduate School of Arts and Sciences, Meguro-ku, Tokyo, Japan; Japan Society for the Promotion of Science, Chiyoda-ku, Tokyo, Japan; Department of Life Sciences, 68394 The University of Tokyo Graduate School of Arts and Sciences, Meguro-ku, Tokyo, Japan; Department of Life Sciences, 13143 The University of Tokyo, Meguro-ku, Tokyo, Japan

**Keywords:** texture, natural image statistics, spatial vision, surfaces/materials

## Abstract

The visual system represents textural image regions as simple statistics that are useful for the rapid perception of scenes and surfaces. What images ‘textures’ are, however, has so far mostly been subjectively defined. The present study investigated the empirical conditions under which natural images are processed as texture. We first show that ‘texturality’ – i.e., whether or not an image is perceived as a texture – is strongly correlated with the perceived similarity between an original image and its Portilla-Simoncelli (PS) synthesized image. We found that both judgments are highly correlated with specific PS statistics of the image. We also demonstrate that a discriminant model based on a small set of image statistics could discriminate whether a given image was perceived as a texture with over 90% accuracy. The results provide a method to determine whether a given image region is represented statistically by the human visual system.

## Introduction

Images of natural environments contain textural regions such as the surface of an object or the ensemble of objects (Gibson, 1979). The primate visual system represents textural information as simple image statistics, and it uses these statistics for rapid recognition of natural images (Motoyoshi et al., 2007; Oliva & Torralba, 2006; Thorpe et al., 1996). However, not all image regions are textures. People see gravel or fabric as a texture, but they do not see single stones or houses as texture. And so what kinds of natural image regions do we perceive as textures? If researchers had a criterion for ‘texture’ images, as opposed to other natural images such as scenes and objects, it would be helpful in investigating the natural mechanisms of statistical visual processing.

A number of theories suggest that the perception of visual texture is determined by the global statistics of the image region (Julesz, 1965; Landy & Graham, 2004). In particular, the Portilla-Simoncelli statistics model (henceforth PS model) accurately predicts the appearance of a natural texture, and it can synthesize a similar texture only by equating image statistics (Portilla & Simoncelli, 2000). The PS model supports the idea that a particular set of global image statistics determines the appearance of individual natural texture. This suggests that ‘texture’ can be defined as an image region whose perception is well described by global image statistics.

The PS model successfully synthesizes the appearance of images normally considered as ‘texture’, but it fails to synthesize ‘non-texture’ images (Portilla & Simoncelli, 2000). Whether or not a natural image is perceived as a texture, then, can be closely related to whether the image is represented by the PS statistics. Furthermore, considering that some image statistics such as the power spectrum differ between images of scenes and textures (e.g., Hansen and Hess, 2006), it is possible that whether an image is perceived as a texture is predictable from the statistics of the image itself. Accordingly, whereas the ‘non-texture’ images such as scenes are assumed to involve higher-order information beyond the image (e.g., PS) statistics, the existence of such higher-order information may be reflected in the image statistics themselves. If this is the case, it is expected that one can predict whether an image will be perceived as a texture by using its image statistics.

The present study examined these possibilities using a variety of natural images. We first measured whether the images were perceived as textures and whether the PS-synthesized images were perceptually similar to the original images. The results showed that the two judgements were very highly correlated (r > 0.9). This robust relationship demonstrates that the perceived ‘texturality’ of an image is closely related to how well it is represented by the image statistics. Next, we identified summary PS statistics that were strongly correlated with the texture/non-texture judgement, and we used these statistics as input to construct a support-vector machine (SVM) that was designed to classify images into texture or non-texture. With only a few statistics, the machine was able to predict whether a given image would be perceived as a texture or not with over 90% accuracy. These results suggest that image statistics can be used to predict whether the human visual system processes a given natural image as a texture. This finding provides a useful tool in the study of texture and ensemble perception, as it operationally defines and objectively selects the natural images that the visual cortex processes as ‘texture’ or ‘ensemble’.

## Method

### Observers

Five naïve paid volunteers and three of the authors took part in the experiment (21–25 years old, mean = 22.4). All observers had normal or corrected-to-normal vision. All the experiments followed the Declaration of Helsinki guidelines and were conducted with permission from the ethics committee for experiments on humans at Graduate School of Arts and Sciences, The University of Tokyo. All observers provided written informed consent.

### Apparatus

Visual stimuli were generated by a PC and displayed on a LCD monitor. Owing to the situation of COVID-19, stimuli were displayed on a LCD monitor (two BENQ XL2720B, three BENQ XL2730Z, BENQ XL2735, SONY PVM A250, and SONY PVM 2541A) set up in the participant's own home. The background mean luminance was in the range of 26–49 cd/m^2^. All monitors had gamma-corrected luminance as calibrated with a colorimeter (ColorCal II CRS) and a frame rate of 60 Hz. The viewing distance adjusted so that the pixel resolution was 0.97 min/pixel. As a result, the size of the uniform background varied among monitors (from 30.7(W) × 17.3(H) to 41.0(W) × 23.0(H) deg).

### Stimuli

**Figure 1.**
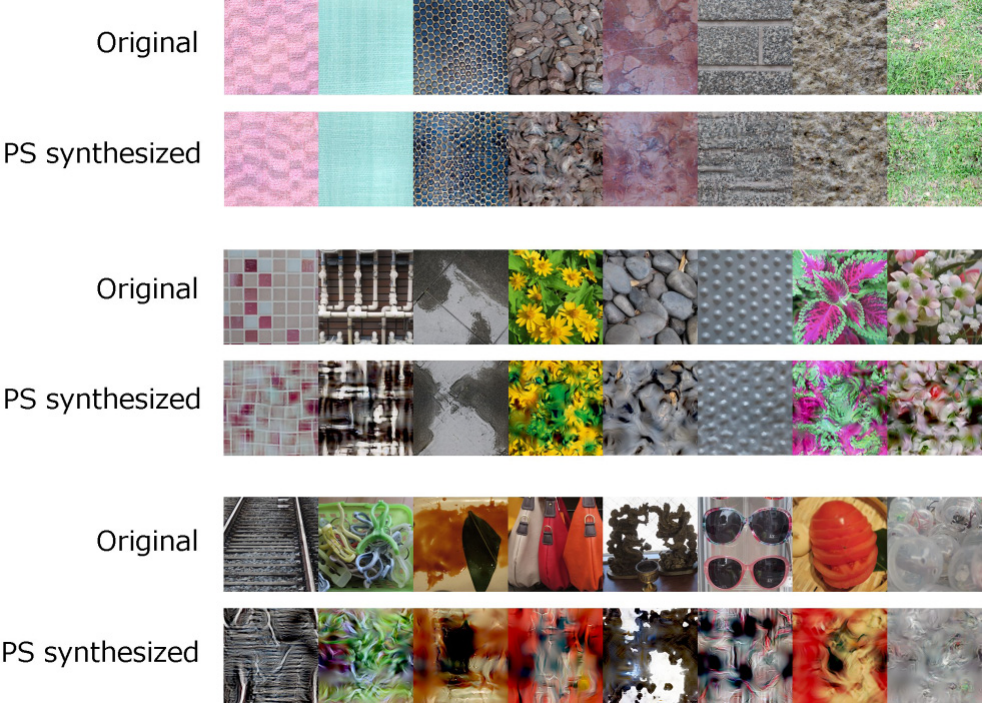
Examples of natural images used in the experiment.

The visual stimuli were 500 natural images (4.1 × 4.1 deg) and their PS-synthesized version (Figure 1). The original images involved various categories such as object groups, scenes, surfaces, etc., which were collected from our own image database and other sites on the internet. The PS synthesis was performed using the original parameter settings in Portilla and Simoncelli (2000) (4 scales, 4 orientations, 7 adjacent pixels, 20 iterations).

### Procedure

Observers performed two different tasks in two separate blocks. In one block, observers freely viewed the original image presented in the center of the display and classified the image either as ‘texture’ or ‘non-texture’. In the other block, observers freely compared the PS-synthesized image with the corresponding original image. The original image was presented randomly either on the left or right side with at a 4-deg eccentricity from the display's center. Participants indicated whether the PS-synthesized image was similar to the original image. In each block, 500 stimuli were presented in a random order. For each observer, at least four responses were collected for each image (in separate trials) and then the proportion of ‘similar’ (or ‘texture’) responses was averaged over observers. We also measured perceptual similarity on a five-point rating scale ranging from “obviously different from the original natural texture” (0) to “nearly the same as the original natural texture” (4).

## Results

**Figure 2.**
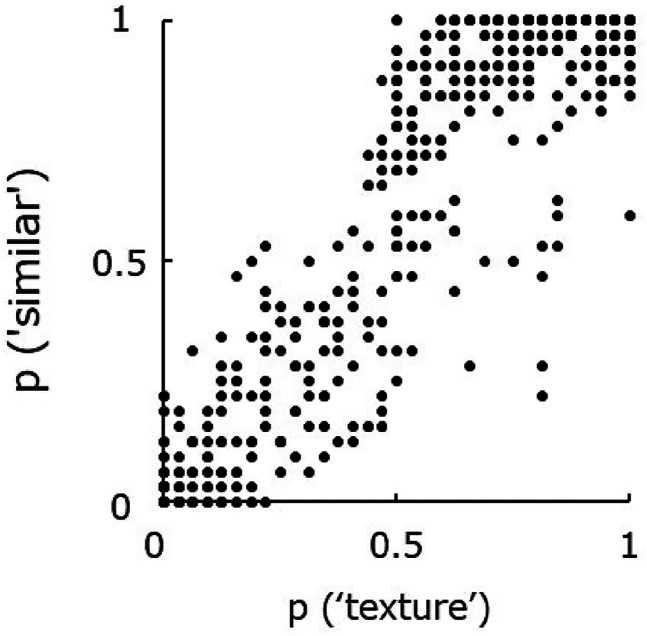
Relationship between the proportion at which the original image was classified as “texture” (horizontal axis) and the proportion at which the PS-synthesized image was perceived to be sufficiently similar to the original image (vertical axis). Each point represents the average of 8 observers.

Figure 2 shows the relationship between the response rate for which the original image was classified as ‘texture’ and the response rate for which the PS-synthesized image was perceived to be similar to the original image. The two measures are strongly correlated (r = 0.92) and indicate that apparent ‘texturality’ is closely related to PS synthesis success or failure: We confirmed that the correlation is not lowered even if max responses (p = 1) were excluded from the analysis (r = 0.91). In other words, images that can be reproduced by PS statistics are perceived as textures, and images that cannot be reproduced are classified as non-textures. We observed a similar pattern of the results using five-point scale rating, as we found that similarity was also strongly correlated with the texturality (r = 0.93).

**Figure 3.**
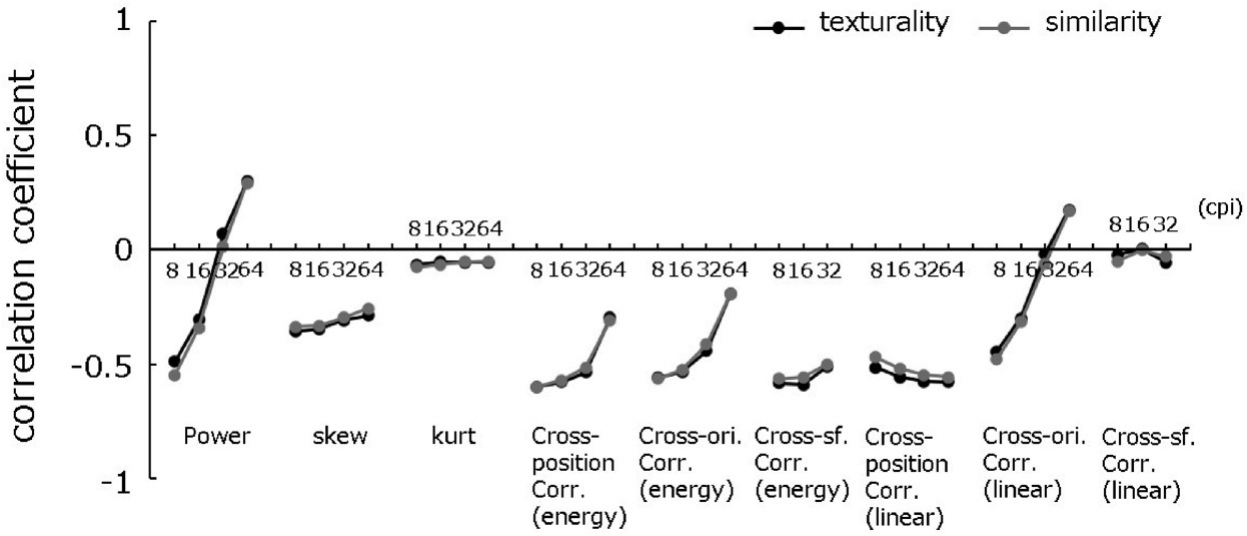
Logistic correlations between PS statistics and judgments of texturality of the image (black) and similarity between synthetic and original image (grey). Each curve shows the results for different classes of summary image statistics plotted as a function of spatial frequency.

Next, in order to examine whether the above data can be predicted by partial PS statistics, we searched for PS statistics that are highly correlated with discriminant data. While the PS statistics of a color image consists of thousands of variables, they can be summarized into a relatively small number of classes; power, skew, and kurtosis at each spatial frequency subbands, and cross-position/orientation/frequency correlations across linear/energy subbands (Portilla & Simoncelli, 2000). We then calculated the average within-class PS statistics for each spatial frequency and summarized them into 34 variables. Figure 3 shows the correlation coefficients between these variables and the discriminant data. These plots show that the observers’ judgments are especially highly correlated with classes of PS statistics, including cross-position correlation in the linear and energy subbands, cross-orientation/scale correlation, and spectral power (at low spatial frequency).

**Figure 4.**
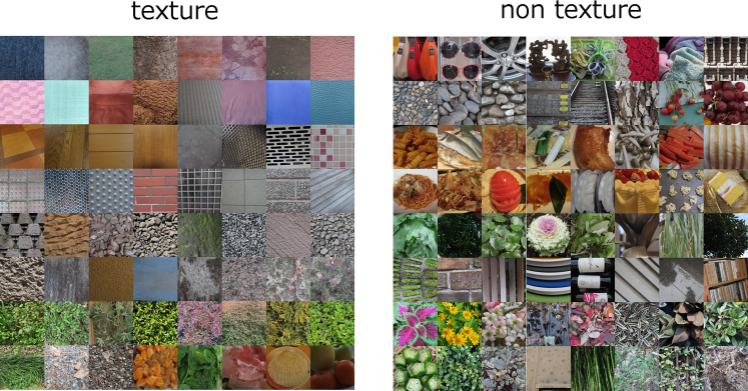
Examples of images classified as ‘texture’ (left) and ‘non-texture’ by the model with six summarized PS statistics.

Finally, we tested whether a support vector machine (SVM) trained with these variables could predict the apparent texturality and the success-failure of PS synthesis. In training the SVM, the data were labeled according to whether the average proportion was higher or lower than 0.5. We employed only the six most highly correlated statistics (cross-position energy correlation at 8 and 16 cpi, cross-frequency energy correlation at 8 and 16 cpi, cross-position linear correlation at 64 cpi, and cross-position linear correlation at 32 cpi (texturality) or cross-orientation energy correlation at 8 cpi (similarity), As a result of the 10-fold cross-validation, we found that 91% of the images were correctly classified as ‘texture’ (Box Constraint: 916.85, Kernel Scale: 0.0543), and 88% of the images were correctly predicted for the success/failure of PS synthesis (Box Constraint: 0.0142, Kernel Scale: 0.0015) when we treated the human judgements as ground truth. Figure 4 shows image samples that the model classified as ‘texture’ (left) and ‘non-texture’. As we further increased the number of statistics considered, the accuracy reached 93% for judgements of texturality and 92% of judgements of similarity.

## Discussion

The present study investigated objective conditions under which natural images are perceived as ‘textures’. Our psychophysical experiments with 500 natural images showed that a natural image's ‘texturality’ correlates very strongly with the perceived similarity of the PS-synthesized image with the original image. These results indicate that human observers generally perceive natural images as ‘textures’ if such images can be successfully synthesized by PS statistics and otherwise classify it as ‘non-texture’ if it cannot. Provided that ‘visual texture’ is defined by perception rather than by physics, our result suggests that PS statistics are virtually perfect in describing the perception of visual textures.

We also found that the ‘texturality’ and ‘perceived PS similarity’ were highly correlated with some of the PS statistics of the images. Ultimately, using machine learning, we showed that a few summarized PS statistics can be used to predict with very high accuracy whether a given natural image will be perceived as a texture. The results of our psychophysical experiments indicate that linear/energy spatial autocorrelation, cross-orientation / frequency energy correlation, low frequency power are especially important. Linear cross-frequency correlation and kurtosis seem to be of little importance in texturality (and similarity) judgements, as partially consistent with the finding of Balas (2006) who showed that linear cross-band correlations have little impact on the discrimination between the PS-synthesized image and the original image in peripheral vision. In the SVM-based classification, we used only a subset of the image statistics that were most highly correlated with observers’ judgments, but the results do not necessarily suggest that the statistics that were left aside are unimportant. In fact, SVM trained using only moment statistics (power, skew, kurtosis) – some of which were highly correlated with texturality judgments – also showed a relatively high performance (88% accuracy for texturality (Box Constraint: 351.74, Kernel Scale: 1.2965), 89% accuracy for similarity (Box Constraint: 9.6800, Kernel Scale: 1.7189) by using 12 variables). The moment statistics are essential parameters in the Heeger-Bergen texture model (Heeger & Bergen, 1995), suggesting that the higher-order statistics used in the PS statistics are not the only ones to play an important role.

Simply put, the fact that a PS-synthesized image is not perceptually similar to the original image suggests that the image contains higher-order information outside of the PS statistical space. In light of this, it may appear strange that PS statistics can predict whether PS synthesis is successful or not. An idea to reconcile this apparent contradiction is that higher-order information is predictable from low-level image statistics. However, it should be noted that the present data do not necessarily suggest that such higher-order information are causally linked to low-level image statistics. Casual observation of non-texture images in Figure 4 suggests that higher-order information may be related to object contours and their shapes or to other types of statistical regularities (e.g. self-similarity) specific to natural scenes (Geisler, 2008; Simoncelli & Olshausen, 2001). The higher-order information could be computed through a neural process as distinct from computation of low-level image statistics. Unfortunately, it is unclear what specific neural representations are elaborated. It is even possible that such representations cannot be described in everyday words such as contours or shapes.

Nevertheless, the present findings provide a simple method which exploits easily computable image statistics to classify whether or not a given image region is perceived as a texture. The method may be useful in psychophysical and neurophysiological studies using natural images to select experimental stimuli or to interpret the results that are also affected by the image category such as surfaces or objects (Motoyoshi et al., 2007; Wiebel et al., 2015).
